# A Century of Medical History in the County of Erie.—1800–1900

**Published:** 1899-01

**Authors:** William Warren Potter

**Affiliations:** Buffalo, N. Y.


### A CENTURY OF MEDICAL HISTORY IN THE COUNTY OF ERIE.—1800–1900.

By WILLIAM WARREN POTTER, M. D., Buffalo, N. Y.

*Pioneer Physicians—Medical Societies—Medical Colleges—Hospitals—Medical Journals—Women Physicians—History of Homeopathy—Medical Officers of the Civil War—Individual Members of the Profession.*

[*Continued from the December edition.*]

### Medical Press of Western New York.

A periodical with the foregoing title was established at Buffalo in August, 1885; at least a number of physicians met during that month and organised a stock company for the purpose of publishing and maintaining a new medical journal, that was subsequently given the name and title of "The Medical Press of Western New York." The stockholders were nearly all physicians residing in Buffalo and the amount subscribed was understood at the time to be about $2,000.

The first number of the magazine appeared in November, 1885, and was in size a small octavo of fifty-two pages. It was edited by Dr. Roswell Park, with the assistance of Dr. Matthew D. Mann, of Buffalo; Dr. Ely Van de Warker, of Syracuse, and Dr. W. J. Herriman, of Rochester. In its salutatory it made the customary appeal to the conventional "kind reader" after the fashion of the rural weekly newspaper, and announced its "policy" in part as follows:

"Let it be the rôle of the large and excellent metropolitan weeklies to serve as advertising mediums for the publishing houses which control them. The Medical Press Association of Western New York has nothing to advertise, neither man, nor clique, nor books; nothing, save the fact that it undertakes the publication of a first-class medical journal, of the profession and for the profession, and deems in this fact it finds its sufficient *raison d'etre.* Kind reader, what think you?"

Dr. Arthur M. Barker was its first business manager, but in July, 1886, Dr. Charles G. Steele succeeded him. Besides the associate editors first mentioned, the following physicians also served in that capacity: Drs. Charles G. Stockton, DeLancey Rochester, J. H. Pryor, of Buffalo, and W. E. Ford, of Utica.

The *Press* continued its publication until June, 1889, when its subscription list, good-will and effects passed into the hands of Dr.

William Warren Potter, who merged it with the BUFFALO MEDICAL JOURNAL.

### MEDICINISCH-CHIRURGISCHES CORRESPONDENZ-BLATT.

For some time previously the German-American physicians residing in Buffalo had discussed the propriety of establishing a medical journal, but it was not put into practical working order until late in 1882. The first number of the magazine, bearing the above title, appeared in January, 1883, under the editorship of Dr. Marcell Hartwig. His associates were Dr. Schwartz, of Vienna; Dr. Reutor, of Berlin; Dr. Engel, of Philadelphia; Dr. Rachel, of New York; Dr. Erichsen, of Detroit; Dr. Proegler, of Fort Wayne, and Drs. Meisburger and Charles Weil, of Buffalo. It was published in the German language and made an excellent appearance. After a year and a half, however, it was discontinued for the lack of adequate pecuniary support.

### V.—WOMEN PHYSICIANS.

The history of medical women in Buffalo and Erie county begins properly with the admission of Mary Blair Moody to the Medical Department of the University of Buffalo in 1874, that being one of the first medical schools to matriculate women. The college records give no account of any action on the part of the faculty concerning the admission of women. They were not denied the privilege by the charter, therefore it was not regarded necessary to take formal action in the matter.

Following her graduation, Dr. Moody practised medicine in Buffalo for several years. Subsequently she went to New Haven, Conn., where she still pursues the practice of her profession, and is known far beyond her immediate circle as a woman interested in all that pertains to the advancement not only of her sex, but of the race.

Prior to Dr. Moody there has been but one reputable woman physician in Buffalo, a Dr. Cook, who with her husband practised medicine according to the teachings of the homeopathic school. These physicians have long since removed from Buffalo, so but little can be learned of Dr. Cook's work here and nothing of her subsequent life.

Dr. Moody had an immediate successor in the college halls in the person of Mary Berkes, who matriculated in 1877. Miss Berkes was born at Williamsville in 1851, and as she had been a teacher before entering college, her preliminary education having been received at Williamsville Academy, she was well up to the present standard of requirements. In 1880 she graduated from the university and immediately began the practice of her profession in Buffalo. During her first year she was the only woman in attendance, but later she had the company of others. She had the good fortune to be invited by Dr. White to attend many of his private operations in gynecology and was well equipped upon graduation. In January, 1886, Dr. Berkes married Dr. S. W. Wetmore, her former preceptor.

Since the Medical Department of the University of Buffalo first admitted women, seventy-two have received their diplomas, many of them with high honors. The complete list of the Alumnæ of the college is as follows:

Katheryn M. Bailey,[1] Buffalo, '89; Gertrude E. Beebe, Buffalo, '91; Ida C. Bender, Buffalo, '90; Alice McL. Ross Bennett, Buffalo, '90; Clara E. Bowen, Buffalo, '92; Marie L. Benoit, Sonyea, '96; Ava M. Carroll, '88; Evangeline Carroll, Buffalo, '93; Jane Wall Carroll, Buffalo, '91; Martha F. Caul, Buffalo, '91; N. Victoria Chappell, Buffalo, '92; Annie May Cheney, Franklinville, N. Y., '98; Georgia Cruikshank, Buffalo, '98. Isabel A. Church, B. S., New York, '93; Salina P. Colgrove, Ph. G., Salamanca, N. Y., '88; Amanda M. Congdon, Cuba, N. Y., '92; Annie B. Culver,[1] Des Moines, Ia., '84; Carro Julia Cummings, B. Ph., Buffalo, '97; Mary I. Denton, Buffalo, '91; Mary E. Dickinson, New York, '90; Louise Downer, Orchard Park, N. Y., '86; Ella May Doyle, East Concord, N. Y., '93; Amelia Dresser, Buffalo, '93; Alice B. Foster (Bryan Mawr), Wakefield, Mass., '91; Maud M. Foy, St. Louis, Mo., '97; Jane North Frear, Buffalo, '94; Maud J. Frye, Buffalo, '92; Anna Wadsworth Hatch, Sauk Center, Minn., '89; Margaret S. Hallick, B. S., Buffalo, '97; Mary J. Hayes, Kushequa, Pa., '97; Jeannette Potter Himmelsbach, Buffalo, '90; Mary M. Huntley, Buffalo, '96; Elizabeth Johnson, New York, '87; Sophia P. Jones, Six Lakes, Mich., '83; Rachel J. Kemball, Buffalo, '84; Regina F. Keyes, Buffalo, '96; Elizabeth M. King, Grand Haven, Mich., '93; Ada C. Latham, Buffalo, '92; Cora Billings Lattin, Buffalo, '94; Emma C. LeFevre, Elmira, N. Y., '92; Elizabeth Fear Leffingwell, Summit, N. J., '88; Caroline Lichtenberg, Buffalo, '98; Emma L. McCray, Lovell's Station, Pa., '91; Marjory J. McPherson, N. Tonawanda, '97. Charlotte E. Mastin, Wellsboro, Pa., '97; Alice Long Mitchell, East Aurora, N. Y., '98; Jennie L. Messerschmidt, Bath, N. Y., '93; Mary Blair Moody, New Haven,

1. Since deceased.

Conn., '76; Helen Kennedy Moorehouse, Buffalo, '85; Nellie Edmunds Murray, Tonawanda, N. Y., '92; Sarah H. Perry, Rochester, N. Y., '82; Alice May Potter, Ithaca, N. Y., '97; Lillian Craig Randall, Buffalo, '91; Marie Ross, ———, '90; Mary E. Runner-Sanford, Buffalo, '81; Marie Cecelia Rotherham, Southport, Eng., '98; Sarah E. Simonet, Croghan, N. Y., '85; Mary Jane Slaight, Rochester, N. Y., '80; Ellen Robert Spragge, Buffalo, '88; Elizabeth M. Squier, Albion, N. Y., '93; Isabella H. Stanley, Dunkirk, N. Y., '83; Edith Winifred Stewart, Hume, N. Y., '98; Anna M. Stewart, Elmira, N. Y., '95; Clara B. Talbot Weidman, Rockport, Me., '90; Marian A. Townley, Ithaca, N. Y., '89; Amelia Earle Trant, Buffalo, '94; Bina Potter Van Denbergh, Buffalo, '83; Stella Cox Venable, Geneseo, N. Y., '88; Carolyn Westlake, Le Roy, N. Y., '97; Loretta E. Wooden, Rochester, N. Y., '—; Frances Weidman Wynds,[1] Brooklyn, N. Y., '91; Mary Berkes Wetmore, Buffalo, '80.

In the spring of 1892, upon recommendation of the medical faculty, the trustees of Niagara University voted to admit women to the medical department of that institution. Anna Earl Hutchinson, of North Evans, was the first woman to avail herself of this privilege. She entered college in the fall of 1892, graduating therefrom in 1895. Dr. Hutchinson has been appointed as woman physician on the medical staff of the Manhattan State Hospital, at New York City. In 1897, Miss Mary O'Malley received the diploma of Niagara University. Dr. O'Malley is now serving on the staff of the Binghamton State Hospital.

In connection with the history of medical women in Buffalo, the work of the Women's Union in securing the appointment of women physicians to state hospitals deserves mention. At the monthly meeting of the protective committee, which the Union held in September, 1889, the president, Mrs. Harriet A. Townsend, first proposed their employment in the state hospitals for the insane. On November 4, 1889, letters were sent to fifty-seven superintendents of hospitals for the insane, asking if they employed women physicians, and whether they would approve of women physicians being put in charge of their own sex. Forty-six answers, from thirty-two different states, were received, and of these, thirty-three were favorable, five were opposed, five were noncommittal, and three not prejudiced. Armed with such approval the directors of the Union, on January 7, 1890, authorised Mrs. Townsend to prepare a bill on the subject.

1. Since deceased.

It was presented in the Assembly by the Hon. Wm. F. Sheehan and in the Senate by the Hon. John Laughlin, January 14. Although in committee the bill had a stormy time, it passed the Assembly, March 27th, with only two negative votes and on April 2d the Senate, with a majority of twenty-six to three. April 27th the bill received the signature of the Governor and became a law.

The first physician to be appointed under the law was Dr. Eleanor McAllister, a graduate of the Syracuse University, who was placed on the medical staff of the Buffalo State Hospital by Dr. J. B. Andrews. Owing to ill health Dr. McAllister resigned in October, 1892, and removed to Southern California, where she has since resided.

Dr. Helene Kuhlman succeeded Dr. McAllister and is the present incumbent. Dr. Kuhlman was born in Hamburg, Germany, in 1869, and received her preliminary education in the schools of that city and in Zurich, Switzerland. In 1887, she came to New York and entered the Woman's Medical College of the New York Infirmary, graduating therefrom in May, 1890. She has served as resident physician or interne in the Nursery and Child's Hospital, Staten Island, in the Babies' Hospital, New York, and was engaged in private practice for a short time at Cleveland, Ohio.

There are now in Buffalo engaged in active practice of medicine between twenty and thirty women. Death has removed from the ranks some who have located here, some have married, while several follow the profession of teaching, two at least with eminent distinction, Dr. Amelia Earle Trant, of the High School, and Dr. Ida C. Bender, supervisor of primary grades. Some of these women have acquired such local fame that mention of their work seems justifiable. This historical sketch, indeed, would be incomplete without something concerning them beyond the mere record of their names.

Electa B. Whipple, daughter of Daniel and Charlotte (Alverson) Whipple, was born at Gowanda, Cataraugus county, N. Y., and received her early education in the common school and the Gowanda High School. Subsequently she entered upon a preparatory classical course for college in Genesee Wesleyan Seminary, at Lima, N. Y., and after completing the same entered Genesee College, located at the same place. After two years spent in Genesee College she entered Syracuse University, from which she graduated in 1874, receiving the degree of A. B., and in 1877 the degree of A. M., from the same university. From the Medical College of Syracuse

University she graduated in 1884, receiving the degree of M. D., and at once entered upon the practice of medicine. On May 1, 1888, she formed a copartnership with Dr. Anna Fiske Crowell, who was her classmate in the medical college and they then located at Buffalo. This relation continued until the death of Dr. Crowell, September 2, 1888. Since that time Dr. Whipple has continued the practice of medicine at Buffalo.

She has been a contributor to the BUFFALO MEDICAL JOURNAL and was a member of the editorial staff of the Woman's Edition of the JOURNAL, published in June, 1896. She is a member of the Alpha Phi Society, of the Buffalo Microscopical Society, of the Physicians' League, of the Medical Society of the County of Erie and of that of the State of New York, and a Fellow of the Buffalo Academy of Medicine.

Dr. Jessie Shepard, perhaps the leading woman of the Homeopathic School, now practising in Buffalo, was born in 1861. Her mother belonged to one of the pioneer families of Buffalo. She was educated at the High School, at that time known as the Central School. In 1884 she entered the office of the late Dr. A. C. Hoxsie as a student of medicine. In 1888 she graduated from the Boston University School of Medicine, serving during her senior year as interne of the Massachusetts Homeopathic Hospital. After five years of practice in Buffalo, Dr. Shepard spent the year 1894 in Europe, chiefly in Schauta's clinic, in Vienna, in the study of gynecology and obstetrics. She is now in active practice in Buffalo. Dr. Shepard is assistant obstetrician at the Buffalo Homeopathic Hospital, and a member of the American Institute of Homeopathy, the Homeopathic Medical Society of the State of New York, the Western New York Homeopathic Medical Society, and the Erie County Homeopathic Medical Society. She is also corresponding secretary and treasurer to the Buffalo Microscopical Society.

Dr. Rose Wilder was born at Akron, Erie county, N. Y., in 1857. She received a high and normal school education, and in 1891 entered the Homeopathic College of Michigan University, graduating therefrom in 1884. She served two years as resident physician in the Industrial Home for Girls, at Adrian, Michigan. Dr. Wilder practised for two years in Akron, and then entered Boston University School of Medicine for post graduate work. In 1889 she began the practice of medicine in Buffalo, where she has since been located. She is assistant obstetrician to the Buffalo Homeopathic Hospital and a member of the Western New York

Homeopathic Medical Society and of the Erie County Homeopathic Medical Society.

Dr. Jane Wall Carroll was born at Paterson, New Jersey, February 20, 1848. She received her early education at Mount St. Vincent Academy on the Hudson, leaving that institution in 1864. She married Peter V. Carroll, of New York, May 13, 1867, and came to Buffalo in 1878. She entered the Medical Department of the University of Buffalo and graduated therefrom in 1891. Following this she took post graduate work at the Polyclinic in New York. Dr. Carroll was one of the pioneers in the Woman's Educational and Industrial Union, having been for five years a vice-president and director. During the early years of her residence in Buffalo she was leading soprano in St. Joseph's Cathedral. Dr. Carroll has been president of the Physicians' League, and a member of the Medical Society of the County of Erie, and a Fellow of the Buffalo Academy of Medicine.

Dr. Carroll is the mother of ten children, the eldest of whom, Evangeline Carroll, graduated from the Medical Department of the University of Buffalo in 1893. Her eldest son, William Carroll, is a graduate of the Buffalo Law School. Her husband, Peter V. Carroll, died in April, 1896.

Maud Josephine Frye was born in Concord, Erie county, N. Y. Her early education was obtained in the district schools of her native town. In 1885 she graduated from Griffith Institute, Springville, N. Y. In the autumn of 1889 she matriculated in the Medical Department of the University of Buffalo, having previously to this studied medicine with Dr. W. A. MacFarlane, of Springville, for one year. In 1892 she graduated from the University with highest honor, being the first woman to attain the distinction in this school. Until May, 1893, Dr. Frye served as interne in the Woman's Hospital at Detroit. Since that time she has practised medicine in Buffalo. She has been clinical instructor in diseases of children in the University Dispensary and is a visiting physician to the babies' ward of the Erie County Hospital and a member of the staff of the Buffalo Woman's Hospital, a member of the Physicians' League, of the Medical Society of the County of Erie, and a Fellow of the Buffalo Academy of Medicine.

There are several other women physicians in Buffalo whose sketches we should like to add did space permit, but we can only mention one other. Dr. Lillian Craig Randall, U. of B. '91, is the only woman physician in the state, we believe, to establish and successfully maintain a hospital for a term of years. The Riverside Hospital must ever be a monument to her energy, and through it will always lie her claim to renown.

### VI.—The Homeopathic Practice of Medicine in Erie County.[1]

Nearly three score years ago, among the settlers that were rapidly filling the thriving village of Buffalo, and establishing its supremacy over its progressive and prominent rival, Black Rock, was a young physician who, filled with professional enthusiasm and encouraged by the prospects of the little town, determined that here should be his future home. He brought with him a good general equipment for the responsible work in which he was about to engage, having taken his degree in the Medical Department of Yale University, but he could not have guessed as he passed through the quiet streets of the village that he was to be the leader in it of a system of medical practice then only just becoming known, or that the little country town was to be one of the great cities of the world.

Dr. N. H. Warner, although not the first in Buffalo to employ homeopathic methods, as Dr. Stephens had preceded him by several years, was yet entitled to be considered the pioneer by reason of his long and important work here and the prominent position which he achieved. He was a quiet, dignified man, reserved in his confidences, but true and loyal to his friends. He had a rarely magnetic presence, a quick insight, together with a firmness and a self-reliance that gave evidence of conscious power. It was not remarkable, therefore, that he rose rapidly in the confidence and in the esteem of his fellow-citizens. He already had made for himself an enviable position in the community, being at this time physician in charge of the Marine Hospital, when he became interested in the new practice which some German physicians had brought from Hahnemann, who was then in the height of his fame in Paris. It soon became evident to him as his studies progressed that a great natural law had been opened up, and believing the apostolic injunction, "prove all things, but hold fast that which is good," he began immediately to make application of his newly acquired knowledge. It was not until 1844, however, eight years after having settled in Buffalo, that we find in his diary, under date of February 6th, the following note: "This day I have made my first purely homeopathic prescription." And then followed a period of

1. For this section the historian is indebted to Dr. F. Park Lewis, who kindly prepared it at his request.

"storm and stress." He was expelled from the County Medical Society, of which he had been a member; professional differences were engendered, with bitter controversies, extending even into the personalities of life; liberty of thought and of action were threatened, and then, with all the pain and suffering that nature seems to have designed as an inevitable accompaniment of such fulfilments, was born in Buffalo the Homeopathic School.

The new practice was destined soon to be tried as by fire. In 1849 cholera came. Dr. Warner's practice had by this time grown to be very large, and during that fateful summer the demands upon his time and strength became too great even for his splendid constitution. His labor was almost incessant day and night, and when finally the scourge had passed it left him exhausted and broken; but his practice was vindicated and the future of homeopathy established. By this time, however, Dr. Warner was no longer obliged to defend the new faith single-handed and alone. Others were led to investigate the homeopathic system through the success which had followed its adoption wherever faithfully tried, and one of the earliest practitioners following Dr. Warner was Dr. George W. Lewis, who in 1849 came with the degree of the University of New York. Dr. Dio Lewis also established himself here, and soon became known throughout the country, through his lectures and writings on hygienic subjects. Dr. P. W. Gray also followed about this time and then came Dr. G. H. Blanchard and Dr. S. Z. Havens, both of whom settled permanently in the then well-grown town.

In 1853 Dr. A. H. Beers, who in many respects was a most remarkable man, came to Buffalo and formed a partnership with Dr. Warner. Dr. Beers was a man liberally educated in the arts as in medicine. He was a graduate of Yale College and of the University of New York. To the culture of a gentleman he added the skill of a trained physician, and the new method of practice, which by this time had a large body of adherents among the most thoroughly representative people of the town, became still more popular. This partnership continued for two years, when Dr. Beers opened a separate office. In 1853 came again an epidemic of cholera, and this time, although the number of homeopathic physicians had increased, the labor which each performed was stupendous.

On the 11th of May, 1856, Dr. A. S. Hinckley began his practice in Buffalo. During that same year Dr. L. M. Kenyon, of Westfield, became Dr. Warner's partner, and in 1859 Dr. A. R. Wright, a former student, became also an associate in the same office.

During these years the Drs. Ehrmann, German physicians of unusual skill, established themselves in Buffalo, and soon had a large and loyal following. They subsequently removed to Cincinnati, where they acheived a national reputation.

By this time a sufficient number of medical men had adopted the newer practice to give the movement a strength that was not inconsiderable and an action was taken which was of critical import in its bearing upon the subsequent medical history of the city.

On December 14, 1859, fifteen members of the homeopathic practice met and formed a society which was to be known henceforth as the Erie County Homeopathic Medical Society. It had for its object "mutual benefit and the advancement of medical science in general."

The first officers chosen for its management were Simon Z. Haven, president; Charles E. Schuch, vice-president; Lorenzo M. Kenyon, secretary; Alvin Shattuch, treasurer; censors, N. H. Warner, Charles E. Schuch, George W. Lewis, Alfred H. Beers and A. S. Hinkley.

The following year, uniting with other similar societies and with representative men from other counties, the New York State Homeopathic Medical Society was organised.

The development of the homeopathic practice from this time on was very rapid. In the early sixties events followed each other in quick succession. The excitement with which the war was ushered in was followed by a suspense like that between the lightning flash and the crash of thunder. Into these intervals came the bulletins from the seat of war, with now and then a call to action. The sentiment of loyalty to the flag, which absorbed personal ambitions, and even stronger interests, in the hearts of many of the bravest men in the country, did not fail to secure its recruits from the medical profession, and among those who early hastened to the front was Dr. Nehemiah Osborne. He returned with honor when the war was over, and for thirty years more continued to fight disease and death in our city, until he himself was vanquished by the last great conqueror.

In 1862 Rollin R. Gregg, M. D., began his practice in Buffalo. Dr. Gregg was a man of intensely strong convictions and one of the most consistent representatives of pure homeopathic methods in America. He believed in the accurately selected similimum, the single remedy, and the attenuated or potentised dose. He rarely used adjuvants, and was exceedingly careful in his sanitary regulations. He had a distinctive, but a large and representative practice, his clientéle extending over the entire country, and he exercised a marked and lasting influence upon the practice in Buffalo.

In 1864, Dr Augustus C. Hoxsie began practice as an associate of his former preceptor, Dr. A. R. Wright, and a few years later, having become firmly established in the esteem of the community, opened an office for himself. He was markedly successful from the first. In a dozen years his practice had reached very large proportions, and before his death it was exceeded in extent and character by that of no other practitioner in Buffalo. Dr. Hoxsie was not a large man, but he had a remarkable personality. Quick and keen mentally, active in his motions, self-possessed and self-controlled, he gained the confidence and esteem of his patients to an unusual degree.

In 1865, Dr. J. W. Wallace was president of the county society, and Drs. H. N. Martin, G. C. Hibbard and Lyman Bedford were among the new members. At a meeting of this organisation in October, 1867, the names of Drs. Hubbard Foster, E. C. Cook and Alexander T. Bull were proposed and accepted for membership. They had come to Buffalo only a short time previous, but they soon acquired large practices and became influential in the school, as in the community, to which they belonged.

The name of Dr. Henry Baethig was also added to the society records about this time, and subsequently that of Dr. George F. Foote. But from 1869 forward the additions to the ranks were too numerous to allow even a bare mention of the names of those who served the cause, and we are obliged to limit ourselves so far as individual mention is concerned strictly to those who, having finished their work among us, are entitled to the distinction of having made the "history" of homeopathic medicine in Buffalo.

Those of us who are still making history, and cannot date our work back farther than a quarter of a century, will have to look to future volumes and future chronicles for a recognition of individual service.

Among those whose claim to our space is sadly undisputed is Dr. S. N. Brayton, whose genial face and bluff, hearty manner will long be remembered by those who counted themselves fortunate as his friends or patients.

Another joyous spirit, whose sudden departure from among us left deep sorrow in the hearts of his brothers in the profession, was Dr. Louis A. Bull. Dr. Bull was one of the brightest and most energetic of the younger men in the school. He had chosen laryngology as his special work, and had already secured broad recognition in this department. His personal characteristics of strength, genuineness and good fellowship, no less than his professional skill, won for him a multitude of friends outside of the profession, who still mourn his early death, which occurred in November, 1894.

In October, 1895, Dr. Abby J. Seymour was accidentally killed. Dr. Seymour was one of the women whose work had given character to homeopathy in Buffalo, and her sad death was a shock to the entire community and an occasion of deep regret.

(*Continued next month.*)

